# Catheter Ablation of Right Ventricular Endocavitary Arrhythmias

**DOI:** 10.1016/j.jacadv.2025.101985

**Published:** 2025-07-17

**Authors:** Ikram U. Haq, Fatima M. Ezzeddine, Nader Al-Shakarchi, Samuel J. Asirvatham, Freddy Del-Carpio Munoz, Abhishek J. Deshmukh, Christopher V. DeSimone, Paul A. Friedman, Gurukripa N. Kowlgi, Malini Madhavan, Peter A. Noseworthy, Suraj Kapa, Konstantinos C. Siontis, Nicholas Y. Tan, Alan Sugrue, Ammar M. Killu

**Affiliations:** a Department of Cardiovascular Medicine, Mayo Clinic, Rochester, Minnesota, USA; b Department of Internal Medicine, Mayo Clinic, Rochester, Minnesota, USA

**Keywords:** cardiomyopathy, catheter ablation, moderator band, premature ventricular contractions, right ventricular arrhythmias, ventricular fibrillation

## Abstract

**Background:**

Right ventricular (RV) endocavitary arrhythmias remain poorly characterized.

**Objectives:**

The purpose of this study was to define the clinical presentation, ablation outcomes, and long-term prognosis of RV endocavitary arrhythmias.

**Methods:**

Among 3,873 patients undergoing ventricular arrhythmia ablation between 2013 and 2025, 53 (1.4%) were included (mean age 45.4 ± 16.9 years, 64% male, mean left ventricular ejection fraction 54 ± 11%).

**Results:**

Forty-three (81%) had structurally normal hearts and 10 (19%) had nonischemic cardiomyopathy, including 7 with premature ventricular contraction (PVC)-mediated cardiomyopathy and 3 with idiopathic nonischemic cardiomyopathy. Ablation indications included PVCs (n = 25), PVC-triggered ventricular fibrillation (VF) (n = 20), and ventricular tachycardia (n = 8). PVC QRS duration independently predicted developing PVC-mediated cardiomyopathy (*P* = 0.02). PVCs-triggering VF had shorter coupling intervals (CIs) (320 [295-358] vs 440 [400-470] ms; *P* < 0.05) and more frequently originated at the lateral moderator band (MB) (*P* = 0.03), where they also had shorter CIs than medial MB PVCs (*P* = 0.01). Ablation targets included the MB (n = 47), anterior papillary muscle (PM) (n = 3), inferior PM (n = 2), and conus PM (n = 1). Postablation increase in sinus rhythm QRS duration (98 [84-102] to 102 [90-114] ms; *P* < 0.01), V1 intrinsicoid deflection (22 [18-27] to 26 [20-95] ms; *P* < 0.01), and new right bundle branch block (15% of patients) did not translate into RV dysfunction or worsening tricuspid valve function. Radiofrequency energy was used in 49 patients, adjunctive cryoablation in 6, and cryoablation alone in 4. At 3.6 (1.6–5.7) years follow-up, 89% achieved clinical success with reduced antiarrhythmic drug use.

**Conclusions:**

RV endocavitary arrhythmias typically occur in structurally normal hearts as focal PVCs. PVCs-triggering VF have shorter CIs and preferentially arise from the lateral MB. Ablation is effective in management.

The right ventricular (RV) endocavitary complex can be a critical site for ventricular arrhythmias, with varied clinical manifestations ranging from benign premature ventricular contractions (PVCs) to PVC-triggered ventricular fibrillation (VF) and ventricular tachycardia (VT).^1^ The internal anatomy of the RV is distinguished by numerous coarse trabeculations and unique endocavitary structures, notably the moderator band (MB) which extends across the RV cavity and inserts toward the base of the anterior papillary muscle (PM) in most patients.^2^ This complex consists of myocardium and an interspersed dense subendocardial network of specialized Purkinje tissue which can provoke arrhythmias.^3^ Given the complex anatomy, catheter ablation of these arrhythmias can be challenging and prior studies examining long-term procedural success are limited and typically involve small patient cohorts.^1^^,^^4^, ^5^, ^6^, ^7^, ^8^, ^9^, ^10^, ^11^ In this study, we expand on the clinical presentation of RV endocavitary arrhythmias and assess both acute and long-term outcomes of ablation at these sites.

## Methods

### Study population and data collection

This study was approved by the Mayo Clinic Institutional Review Board, and the data supporting its findings are available upon reasonable request. We used an electronic database maintained by Mayo Clinic Cloud, which employs natural language processing for data retrieval, to identify all catheter ablations of ventricular arrhythmias performed at Mayo Clinic between 2013 and 2025. Only patients who had previously consented to the use of their medical records for research and underwent ablation targeting the RV MB and PMs were included. We reviewed all available electronic health records, electrocardiograms (ECGs), transthoracic echocardiograms, cardiac monitor tracings, cardiac device interrogations, cardiac magnetic resonance imaging (CMR) imaging, and procedural details of eligible patients. The time to intrinsicoid deflection in lead V_1_ for RV activation time was measured as previously described.^12^

### Electrophysiological study, mapping, and ablation

The index procedure was defined as the first electrophysiological study in which RV endocavitary structures were targeted for ablation at our institution. All procedures were performed in accordance with Mayo Clinic institutional guidelines. Conscious sedation was preferred over general anesthesia. Vascular access was established using ultrasound guidance, and intracardiac multipolar catheters were introduced. Catheter contact with endocavitary structures was confirmed using intracardiac echocardiography, loss of local electrograms (EGMs) and occasionally with loss of pace capture. An electroanatomical 3-dimensional mapping system (CARTO, Biosense Webster; and EnSite, Abbott) was utilized. In the absence of spontaneous arrhythmias, provocation maneuvers, including isoproterenol (2-20 μg/min) administration and pacing, were performed at the discretion of the proceduralist. When arrhythmias were present or inducible, detailed activation mapping was the preferred strategy, with pace mapping used as an adjunct when necessary. Ablation sites on the MB were classified into 3 anatomical regions using intracardiac echocardiography and electroanatomic mapping: 1) medial: RV septal insertion; 2) body: mid-portion traversing the RV cavity; and 3) lateral region: RV free-wall insertion (Central Illustration). The coupling interval (CI) of a PVC was defined as the time interval between the beginning of the preceding QRS to the start of the PVC.^13^ The choice of ablation energy—radiofrequency (RF) ablation, cryoablation, or a combination of both—was determined by the operator. Postablation testing was performed in all cases. Following ablation, patients were monitored on telemetry, and any procedural complications were systematically recorded. Antiarrhythmic drug (AAD) therapy postablation was at the operator's discretion.

**Central Illustration:**
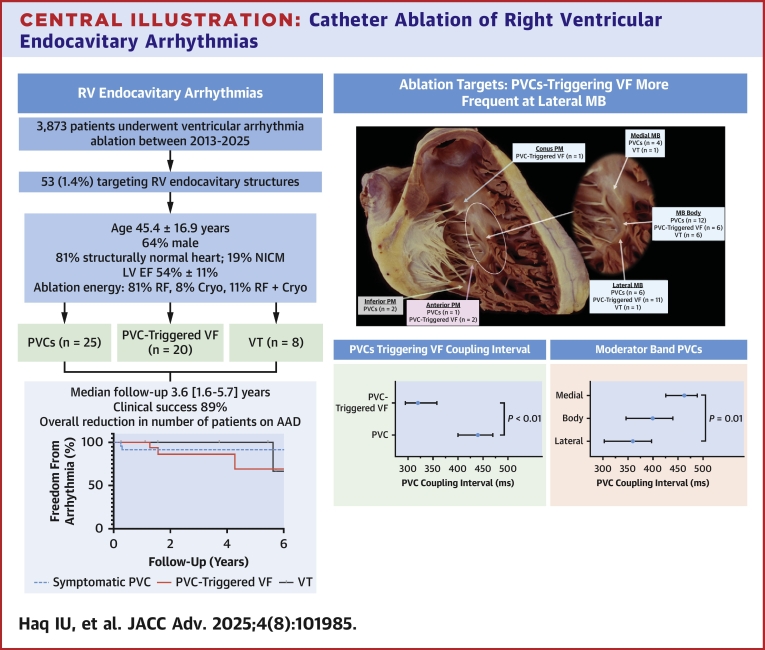
Catheter Ablation of Right Ventricular Endocavitary Arrhythmias AAD = antiarrhythmic drug; LVEF = left ventricular ejection fraction; NICM = nonischemic cardiomyopathy; RF = radiofrequency; RV = right ventricular; other abbreviations as in Figures 1 and 2.

## Clinical outcomes and follow-up

Acute procedural success was defined as the noninducibility of the targeted arrhythmia following a waiting period of at least 30 minutes postablation with testing where appropriate. After the procedure, patients were followed up at 1-, 3-, 6-, and 12-month intervals, with subsequent follow-ups determined at the discretion of the medical team. During these visits, a 24-hour Holter monitor, 12-lead ECG, and interrogation of any implantable cardiac devices (such as loop recorders, pacemakers, or defibrillators) were performed and reviewed. Long-term clinical success was defined based on the clinical indication for ablation (Table 1). Preablation, postablation, and last follow-up ECGs and transthoracic echocardiograms were reviewed for sinus rhythm QRS duration, right bundle branch block (RBBB), cardiac function, and valvular heart disease.

**Table 1:** Definitions of Clinical Success at Last Follow-Up

Symptomatic PVC	Absence of clinical symptoms at last clinical follow-up and a reduction of PVC burden by >80%
PVC-mediated cardiomyopathy	Absence of clinical PVC on follow-up cardiac ambulatory monitoring
PVC-triggered VF	Freedom from any appropriate ICD therapy
VT	Freedom from sustained VT or any appropriate ICD therapy

ICD = implantable cardioverter-defibrillator; PVC = premature ventricular contraction; VF = ventricular fibrillation; VT = ventricular tachycardia.

### Statistical analysis

Continuous variables are reported as mean ± SD if normally distributed or median with IQR if skewed. Normality was assessed using the Shapiro-Wilk test and visual inspection of histograms. Group comparisons were performed as follows: for paired continuous data, we used the paired *t*-test for normally distributed differences or the Wilcoxon signed rank test for non-normally distributed differences; for unpaired continuous data, we employed the Student's *t*-test for normally distributed variables or the Mann-Whitney *U* test for non-normally distributed variables; and for categorical variables, we applied the chi-square test or Fisher exact test when expected cell counts were <5. For comparisons of 3 independent groups, parametric one-way analysis of variance was used when data met both normality (Shapiro-Wilk *P* ≥ 0.05) and homogeneity of variance (Levene's test *P* > 0.05) assumptions. When these assumptions were violated, the nonparametric Kruskal-Wallis test was employed. Univariate logistic regression identified potential predictors of PVC-mediated cardiomyopathy and variables with *P* < 0.05 in univariate analysis were included in a multivariable logistic regression model. All tests were 2-sided, with statistical significance set at *P* < 0.05. Analyses were performed using GraphPad Prism version 10.4.1 (GraphPad Software).

## Results

### Baseline clinical characteristics

Of the 3,873 patients who underwent catheter ablation for ventricular arrhythmias at our institution between 2013 and 2025, 53 (1.4%) met our inclusion criteria. Baseline clinical characteristics are summarized in Table 2. The mean age at the index ablation was 45.4 ± 16.9 years and 64% (34/53) were male. Mean body mass index was 29.3 ± 5.60 kg/m^2^. The mean baseline left ventricular ejection fraction (LVEF) was 54 ± 11%. At the time of ablation, 43 patients (81%) had structurally normal hearts and 10 patients (19%) had nonischemic cardiomyopathy (NICM). Among the 10 NICM patients, 7 had PVC-mediated cardiomyopathy and 3 had idiopathic NICM.

**Table 2:** Baseline Clinical Characteristics (N = 53)

Age at index ablation (y)	45.4 ± 16.9
Male	34 (64%)
BMI (kg/m^2^)	29.3 ± 5.60
Hypertension	18 (34%)
Hyperlipidemia	11 (21%)
Type 2 diabetes	1 (2%)
Atrial fibrillation	15 (28%)
Substrate	
Structurally normal heart	43 (81%)
NICM	10 (19%)
TTE prior to index ablation	53 (100%)
LVEF (%)	54 ± 11
LAVI (mL/m^2^)	31 ± 10.0
RVSP (mm Hg)	30 ± 6
Normal RV size	31 (58%)
Mild RV enlargement	17 (32%)
Moderate RV enlargement	4 (8%)
Severe RV enlargement	1 (2%)
CMR prior to index ablation	30 (57%)
RV EF (%)	49 ± 12
Delayed gadolinium enhancement in RV endocavitary complex	1

Values are mean ± SD or n (%).

BMI = body mass index; CMR = cardiac magnetic resonance imaging; EF = ejection fraction; LAVI = left atrial volume index; LVEF = left ventricular ejection fraction; NICM = nonischemic cardiomyopathy; RV = right ventricular; RVSP = right ventricular systolic pressure; TTE = transthoracic echocardiogram.

**Figure 1:**
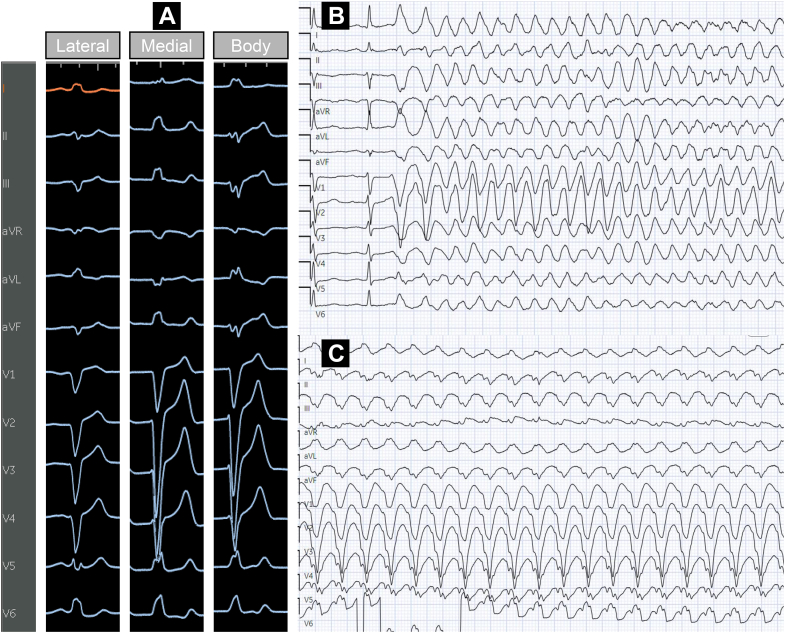
**Example Tracings of Moderator Band Arrhythmias in our Study Population** (A) Representative 12-lead ECGs of PVCs ablated from the lateral, body, and medial moderator band, (B) a PVC with a short coupling interval degenerating into polymorphic VT which was ablated at the lateral junction of the moderator band complex and (C) sustained symptomatic VT ablated from the body of the moderator band. ECG = electrocardiogram; PVC = premature ventricular contraction; VT = ventricular tachycardia.

The clinical presentation of arrhythmias in the cohort varied and were grouped into 3 categories: PVCs in 25 patients (47%), PVC-triggered VF in 20 patients (38%), and VT in 8 patients (15%) (Table 3). Example ECG tracings for the 3 cohorts are depicted in Figure 1. The QRS complex of the clinical arrhythmia showed an rS or QS pattern in lead V_1_, consistent with a left bundle branch block pattern, and a late R-wave precordial transition in lead V_4_ (n = 5), V_5_ (n = 34), or V_6_ (n = 9). During ablation, a change in exit site was noted in 49% of cases. CI were predominantly fixed with minor fluctuations (±10-20 ms) observed and in PVC cases, the CI was found to be independent of baseline HR (r = 0.11; *P* = 0.42). The CI of the PVC in the PVC cohort was 440 (IQR: 400-470) ms and 320 (IQR: 295-358) ms in the PVC-triggered VF cohort (*P* < 0.05). A conduction system signal was noted at the site of ablation in 27 (51%) patients.

**Table 3:** Procedural and Follow-Up Characteristics Grouped by Clinical Presentation

	PVCs (n = 25)	PVC-Triggered VF (n = 20)	VT (n = 8)	*P* Value
Age at index ablation (y)	50.3 ± 17.1	35.3 ± 13.4	54.3 ± 14.1	<0.001
Substrate				
Structurally normal heart	18 (72%)	20 (100%)	5 (62.5%)	0.01
NICM	7 (28%)	0 (0%)	3 (37.5%)	0.01
Enlarged RV of TTE	10 (40%)	8 (40%)	4 (50%)	0.89
PVC burden prior to ablation (%)	15 (9-22)	1 (1-3)	1 (1-1)	<0.001
PVC burden at last follow-up (%)	2 (1-3)	1 (1-1)	1 (1-1)	0.02
QRS duration of clinical arrhythmia (ms)	157 ± 28	156 ± 27	145 ± 34	0.67
Mapping				
Pace mapping only	1 (4%)	6 (30%)	1 (13%)	0.03
Activation ± pace mapping	24 (96%)	14 (70%)	7 (87%)	
Pre-QRS local ventricular electrogram (ms)	35 ± 11	34 ± 11	31 ± 16	0.75
Conduction system signal at the ablation sites	8 (32%)	15 (75%)	4 (50%)	0.006
Ablation energy used				
RF	21 (84%)	14 (70%)	8 (100%)	0.22
Cryoablation	1 (4%)	3 (15%)	0 (0%)	
RF and cryoablation	3 (12%)	3 (15%)	0 (0%)	
New RBBB post index ablation	2 (8%)	2 (10%)	4 (50%)	0.02
Required a 2nd ablation	3 (12%)	4 (20%)	0 (0%)	0.36
Follow-up duration from last ablation (y)	5.2 (2.1-6.5)	2.5 (0.6-3.5)	4.6 (1.5-6.3)	0.01
Success at last follow-up	23 (92%)	17 (85%)	7 (88%)	0.79

Values are mean ± SD or n (%).

RBBB = right bundle branch block; RF = radiofrequency; other abbreviations as in Tables 1 and 2.

### Premature ventricular contractions

In the 25 patients with PVCs, the primary indication for ablation was palpitations refractory to medical therapy (n = 18) and PVC-mediated cardiomyopathy (n = 7). All patients with PVC-mediated cardiomyopathy were male and PVC QRS duration was an independent predictor of developing cardiomyopathy (OR: 1.05; 95% CI: 1.01-1.10; *P* = 0.02) (Table 4). Nine (36%) patients had undergone previous PVC ablations, with 3 cases targeting the RV endocavitary structures. CMR was performed in 14 (56%) patients prior to ablation, and delayed gadolinium enhancement in the RV was only observed in 1 (4%) patient who had previous ablation in the region.

**Table 4:** Univariate and Multivariate Analysis of Predictors of Developing PVC-Mediated Cardiomyopathy

	PVC-Mediated Cardiomyopathy (n = 7)	No PVC-Mediated Cardiomyopathy (n = 18)	Univariate		Multivariate	
			Odds Ratio (95% CI)	*P* Value	Odds Ratio (95% CI)	*P* Value
Age at index ablation (y)	58.0 ± 19.3	46.7 ± 15.5	1.05 (0.99-1.11)	0.09		
BMI (kg/m^2^)	30.5 ± 5.7	27.7 ± 5.3	1.11 (0.94-1.31)	0.22		
Atrial fibrillation	2 (29%)	5 (28%)	1.04 (0.15-7.08)	1.00		
Male (%)	7 (100%)	10 (56%)	∞ (N/A)^a^	a	∞ (N/A)^a^	a
PVC burden (%)	21 ± 11	15 ± 12	1.05 (0.97-1.14)	0.21		
PVC QRS duration (ms)	181 ± 26	146 ± 22	1.07 (1.02-1.13)	0.004	1.05 (1.01-1.10)	0.02

Values are mean ± SD or n (%) unless otherwise indicated.

Abbreviations as in Tables 1 and 2.

a Males had complete separation (all PVC-mediated cardiomyopathy patients were male).

**Figure 2:**
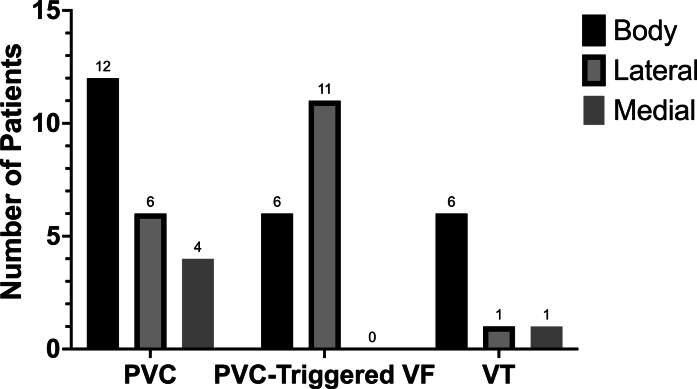
**Site of Ablation on the Moderator Band Complex** Data presented as raw counts (n = 47 total observations) and group comparisons were made using Fisher exact test (*P* = 0.03). VF = ventricular fibrillation; other abbreviations as in Figure 1.

During the index procedure, activation mapping was performed in 24 patients (96%) and the earliest pre-QRS EGM at the site of ablation was recorded at 35 ± 11 ms. Pace-mapping alone was used in 1 patient with a 97% match between the paced and clinical PVC morphology. RF ablation was used in 24 patients and 3 cases used cryoablation adjunctively. Cryoablation alone was used in 1 patient. The primary ablation targets included the MB (n = 22), anterior PM (n = 1), and inferior PM (n = 2). Ablation sites on the MB included the body (n = 12), lateral (n = 6), and medial aspect (n = 4) (Figure 2). In all cases, the clinical PVC was noninducible by the end of the procedure with no acute complications. Four patients (16%) experienced recurrence of the clinical PVC 0.3 (IQR: 0.23-0.55) years after index ablation. One patient died 3 years later from end-stage heart failure. The remaining 3 patients underwent repeat ablation targeting the same focus and at the last follow-up, 2 of the 3 patients had no clinical recurrence.

**Figure 3:**
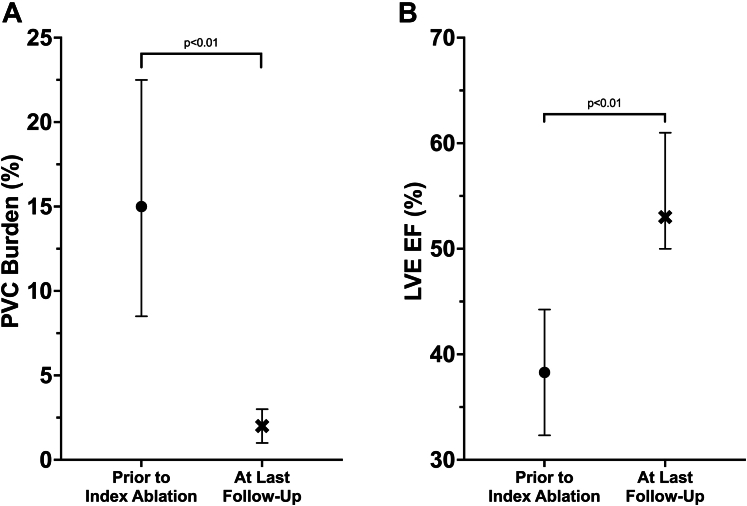
**Changes in Premature Ventricular Contraction Burden and Left Ventricular Ejection Fraction** (A) Premature ventricular contraction (PVC) burden in the 25 patients referred for PVC ablation decreased from 15% (IQR: 9%-22%) prior to the index ablation to 2% (IQR: 1%-3%) at the last clinical follow-up (*P* < 0.01); data displayed as median (IQR). (B) Left ventricular ejection fraction (LVEF) improved from 38% ± 6% to 55% ± 6% in the 7 patients with PVC-mediated cardiomyopathy after catheter ablation (*P* < 0.01); data displayed as mean ± SD.

The 25 patients with PVCs were followed for a median of 5.2 (IQR: 2.1-6.5) years after their last ablation and PVC burden decreased from 15% (IQR: 9%-22%) to 2% (IQR: 1%-3%) (*P* < 0.01). In the 7 patients with PVC-mediated cardiomyopathy, LVEF improved from 38% ± 6% to 55% ± 6% (*P* = 0.03) (Figure 3).

### PVC-triggered VF

**Figure 4:**
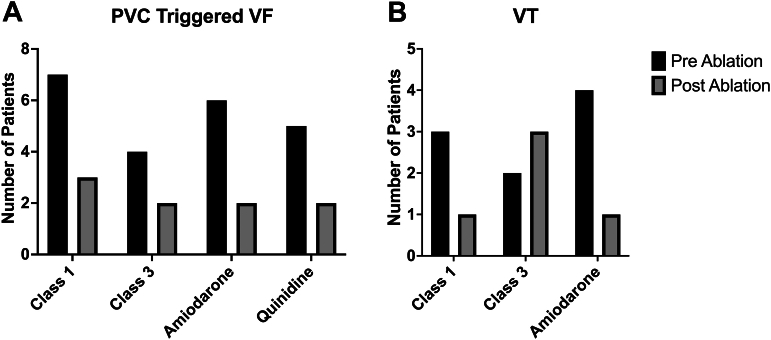
**Antiarrhythmic Therapy** The distribution of antiarrhythmic therapy prior to index ablation and at the last clinical follow-up in patients referred for ablation of PVC-triggered VF (A) and VT (B). Abbreviations as in Figures 1 and 2.

All 20 patients in this subgroup had secondary prevention implantable cardioverter-defibrillators (ICDs) implanted. The mean age at the first episode of VF was 33.1 ± 13.0 years and all patients had preserved biventricular function (mean LVEF 57.8% ± 5.3%). Fifteen patients (75%) underwent comprehensive genetic testing—variants of uncertain significant were noted in 6 patients (LMNA [T655NfsX49], SCN5A [E1240Q], Alpha-protein kinase 3 [p.MET906_Gly911dup], SCN9A [c.5330GC], CACNB2 [g.18789874CT], and KCNQ1 [p.R397W]). Five patients (25%) underwent a procainamide challenge, which yielded negative results. Baseline ECGs showed no evidence of J-wave syndrome, early repolarization, or Brugada syndrome in any patient. Seventeen patients (85%) had CMR performed prior to ablation, revealing delayed gadolinium enhancement in 2 patients (nonspecific mid-myocardial enhancement in the inferolateral basal left ventricle and subendocardial basal RV septum). The indication for ablation in all 20 patients was short-coupled PVC-triggering VF and appropriate ICD therapies. A mean of 1.4 ± 1.1 AADs were trialed prior to ablation, including class I (n = 7), class III (n = 4), amiodarone (n = 6), and quinidine (n = 5) (Figure 4).

During the index procedure, the clinical PVC was spontaneously present in 17 patients (85%). The earliest pre-QRS EGM at the site of ablation of the clinical arrhythmia was recorded at 34 ± 11 ms. RF ablation energy was used in 17 patients, with adjunctive cryoablation used in 3 patients. In 3 patients, cryoablation alone was employed. The primary ablation targets included the MB (n = 17), anterior PM (n = 2), and conus PM (n = 1). Ablation sites on the MB included the body (n = 6) and lateral aspect (n = 11) (Figure 2). In all cases, the clinical PVC was noninducible at the conclusion of the procedure with no procedural complications. Four patients (20%) required repeat ablation 0.59 (IQR: 0.13-1.83) years after their index procedure.

Patients were followed for a median of 2.5 (IQR: 0.6-3.5) years after their last procedure and clinical success (no appropriate ICD therapies) was achieved in 17 patients (85%).

The 3 unsuccessful cases included 2 patients who received 1 appropriate ICD shock for VF during follow-up and 1 patient who developed recurrent ICD shocks despite a repeat ablation and a left-sided sympathectomy who has been doing well since being started on flecainide. At the last clinical follow-up, 45% (9/20) of patients were maintained on AADs, including class I (n = 3), class III (n = 2), amiodarone (n = 2), and quinidine (n = 2).

### Ventricular tachycardia

The indication for ablation in this subgroup was episodes of sustained symptomatic VT in 5 patients, PVC-triggered VT during exercise ECG in 1 patient, and VT storm in 2 patients. The mean age at index ablation was 54.3 ± 14.1 years, 5 (63%) patients had structurally normal hearts without a prior cardiac diagnosis and 3 (38%) patients had NICM with reduced ejection fraction. Cardiac magnetic resonance imaging (CMR) was performed in 7 patients, revealing delayed gadolinium enhancement in 2 patients (basal mid-myocardial septal enhancement and at the RV insertion point). A mean of 1.6 ± 0.9 AADs were trialed prior to ablation, including class I (n = 3), class III (n = 2), and amiodarone (n = 4) (Figure 4).

During the index procedure for the 6 elective VT cases, the clinical VT was induced with ventricular extrastimuli and/or burst pacing in 4 patients, while it could not be induced in 2 patients. In the 2 emergent VT storm cases, 1 patient was in incessant VT during the procedure, while the other patient’s VT was induced with ventricular programmed stimulation. The cycle length of the clinical VT was 378 ± 136 ms, and RF ablation alone was used in all cases targeting the MB. All ablations were acutely successful and after a median follow-up of 4.6 years (IQR: 1.5-6.3 years), 1 patient received appropriate ICD therapies for sustained VT 5.2 years after ablation and 5 (63%) patients remained on AADs, including class I (n = 1), class III (n = 3), and amiodarone (n = 1) (Figure 4).

## Long-term clinical outcomes

**Figure 5:**
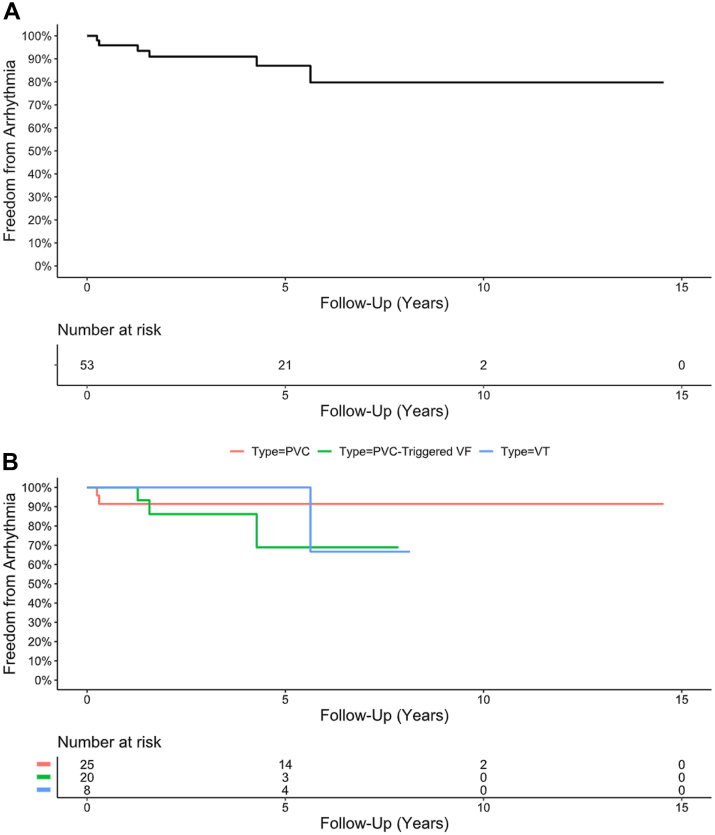
**Freedom From Right Ventricular Endocavitary Complex Arrhythmias Postablation** (A) Cumulative freedom from right ventricular endocavitary complex arrhythmias following index ablation and (B) freedom from arrhythmias stratified by clinical presentation (*P* = 0.49). Abbreviations as in Figures 1 and 2.

After a median follow-up of 3.6 (IQR: 1.6-5.7) years, clinical success was achieved in 47 patients (89%) with an overall reduction in the number of patients remaining on AAD therapy (Figure 5). Sinus rhythm QRS duration increased from 98 (IQR: 84-102) ms prior to ablation to 102 (IQR: 90-114) ms after ablation (*P* < 0.01) and time to intrinsicoid deflection in lead V1 during sinus rhythm increased from 22 (IQR: 18-27) ms prior to ablation to 26 (IQR: 20-95) ms after ablation (*P* < 0.01). 8 (15%) patients developed a new RBBB post index ablation and the MB was targeted in all cases. One (2%) patient developed new mild tricuspid regurgitation. No patients developed long-term sequelae of RV dysfunction or RV failure.

## Discussion

We report our contemporary institutional experience on the ablation of RV endocavitary arrhythmias and the key findings of this study are:

1) RV endocavitary arrhythmias accounted for 1.4% of ventricular ablation cases and the majority (85%) occurred in structurally normal hearts.
2) Focal PVCs were the most common electrophysiological manifestation (85% of cases), of which 44% triggered VF.
3) PVCs-triggering VF had shorter CI (320 [IQR: 295-358 vs 440 (IQR: 400-470)] ms in isolated PVCs; *P* < 0.05) and most commonly arise at the lateral MB.
4) Catheter ablation using RF, cryoablation or a combination of both were effective, though redo procedures were occasionally required.
5) Postablation there was a modest increase in QRS duration and RV activation time and RBBB developed in 15% of patients. This did not translate to clinically significant RV dysfunction or worsening tricuspid valve function.

### RV embryology and anatomy

The RV's unique embryological development and anatomy underlie its arrhythmogenic potential. Unlike the LV which originates from the “first” heart field, the RV is derived from progenitor cells of the anterior (“second”) heart field and features coarse trabeculations forming endocavitary structures like the MB.^14^ These develop through signaling pathways such as Notch and Neuregulin-1 which simultaneously pattern both trabeculae and Purkinje networks.^15^ The resulting subendocardial conduction system and its variable distal ramifications within RV endocavitary structures create a substrate for phase 3 re-entry and focal triggers.^16^ This anatomical and structural variability impacts mapping and ablation targets.^17^

### RV endocavitary PVCs

In our series, focal PVCs were the most common arrhythmia manifestation and 15% had PVC-mediated cardiomyopathy. Cardiomyopathy was better predicted by PVC QRS duration rather than PVC burden, supporting the paradigm that wider PVCs—likely originating farther from the native conduction system—cause greater LV dyssynchrony and dysfunction.^18^ Prior clinical studies have also demonstrated that RV PVCs can develop cardiomyopathy at a lower burden than PVCs from the LV.^19^ As one may expect, elimination of such PVCs result in improvement in cardiac function as was demonstrated in our study. In one case, exercise was a trigger for developing VT and whether exercise is a precipitant of MB VT remains unclear and open for further investigation.

### PVC-triggered VF

**Figure 6:**
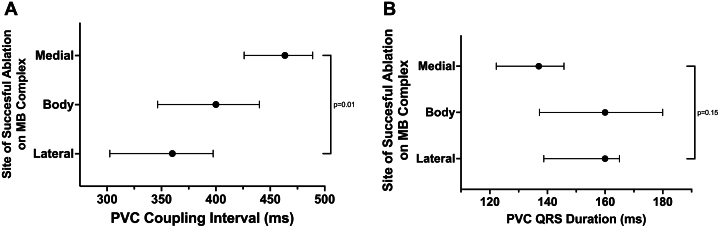
**Coupling Interval and QRS Duration of Premature Ventricular Contractions** (A) Coupling interval and (B) QRS duration of premature ventricular contractions (PVCs) originating from the moderator band stratified by site of ablation. Data presented as median (IQR). Group comparisons were made using the Kruskal-Wallis test. MB = moderator band.

**Figure 7:**
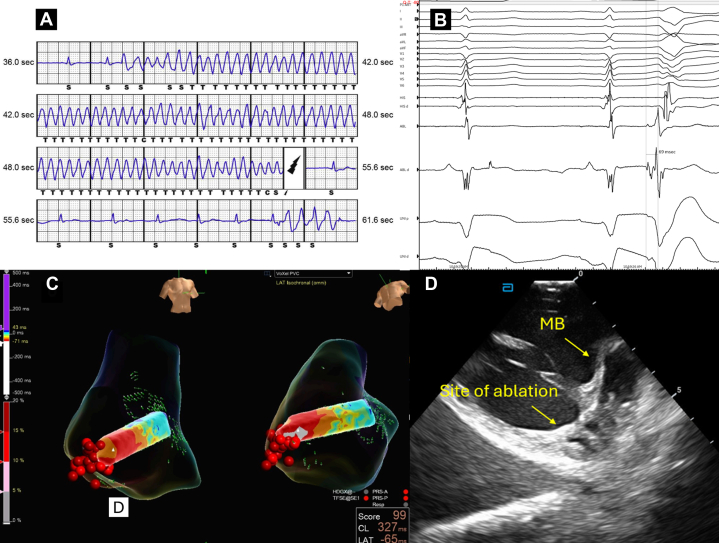
**Example Case** Example case of a 45-year-old gentleman with a history of an out-of-hospital cardiac arrest with documented VF who had recurrent appropriate ICD shocks (A). He had nonobstructive coronary artery disease with a structurally normal heart with focal nonspecific delayed gadolinium enhancement noted at the right ventricular insertion site and a variant of uncertain significance in alpha-protein kinase 3. He underwent ablation for PVC-triggered VF with earliest pre-QRS local ventricular electrogram (69 ms) (B) in the lateral aspect of the moderator band (C). Radiofrequency ablation followed by cryoablation was used with intracardiac echocardiography depicting the site of ablation (D). He was continued on flecainide 100 mg orally twice daily for 3 months postablation without clinical recurrence. ICD = implantable cardioverter-defibrillator; VF = ventricular fibrillation; other abbreviations as in Figures 1 and 6.

Idiopathic VF is a spectrum of concealed arrhythmogenic conditions and short-coupled PVCs triggering VF is an important subcategory. Our study confirms the critical role of PVC CI as a predictor of malignant potential, while revealing a novel anatomical predilection: RV endocavitary PVCs-triggering VF have a preferential localization to the lateral MB. This is likely a reflection of this region's distinct anatomical and electrophysiological properties which aligns with the concept of Purkinje-myocardial discontinuity as an arrhythmogenic mechanism.^20^ Prior studies have demonstrated that Purkinje fibers distal to their encasing insulation are particularly vulnerable to “gating mechanism” failures, where altered action potential gradients permit short-coupled PVC arrhythmogenesis.^21^ The lateral MB has a thinner myocardial coverage with reduced insulation creating more Purkinje-myocardial junctions that are susceptible to enhanced automaticity and re-entry.^16^^,^^17^ Our study also found that MB PVCs tended to have shorter CI in the lateral MB also supporting this hypothesis (Figure 6). Figure 7 highlights an example case of a PVC-triggering VF which was ablated from the lateral MB. These observations have clinical implications. When activation mapping of PVC-triggered VF that is suspected to arise from the RV MB is limited by infrequent PVCs, targeted substrate modification of the lateral MB is reasonable. While new RBBB can develop, our study did not find this led to clinically significant RV dysfunction or worsening tricuspid valve function.

RV PVCs which triggered VF showed an intermediate QRS duration (156 ± 27 ms) compared to the characteristically narrow QRS (<120 ms) of left Purkinje-mediated VF. This is likely related to the less extensive Purkinje arborization of the RV compared to the LV.^22^ This electrophysiological distinction may paradoxically increase arrhythmogenicity by enhancing repolarization dispersion. The transient outward current (I_to_) has been implicated in Purkinje early after depolarizations and if severe spatial dispersion of repolarization exists between the Purkinje fibers and surrounding ventricular myocardium, close-coupled PVCs can lead to VF through phase 2 re-entry.^23^ Patients with PVC-triggered VF had infrequent PVCs prior to and after ablation. In addition to PVC trigger ablation as a one potential reason for therapeutic success, this finding raises the possibility of Purkinje network modification precluding the maintenance of VF.^24^

## Ablation energy

Our study reaffirmed the hypothesis that in select patients, trigger ablation for VF is an effective catheter ablation strategy in preventing VF recurrence. The optimal ablation energy source remains a point of controversy with recent studies suggesting cryoablation is more successful as it overcomes issues related to catheter stability.^4^ However, in our study, RF ablation was the most commonly used initial ablation strategy and was still associated with a high likelihood of success. Each modality has its own merit: while cryoablation catheters have better stability during ablation and less automaticity, RF catheters are more steerable and allow creation of electroanatomic activation maps. While there is limited experience in use for RV endocavitary structures, pulsed field ablation has emerged as an ablation technique and preclinical swine studies have demonstrated a lattice-tip catheter able to deliver biphasic monopolar pulsed field ablation can lead to transmural ablation of the MB.^25^

### Study limitations

The results of this study are best interpreted in the context of its limitations. First, this is an observational, retrospective study from a single institution and the patients included are subject to referral and selection bias. Second, a natural language processing database was used to identify patients and inherent variability in electronic health record documentation could have led to missed eligible patients. Third, the retrospective nature precluded systemic assessment of various triggers including exercise. Fourth, although CMR and genetic testing was performed in a subset of patients, the lack of universal protocols may obscure underlying associations. Fifth, the choice of ablation energy and use of AAD were operator dependent.

## Conclusions

RV endocavitary arrhythmias typically occur in structurally normal hearts and most commonly present as focal PVCs. PVCs-triggering VF have shorter CIs and preferentially arise from the lateral MB. Catheter ablation using RF, cryoablation, or a combination of both is effective, though redo procedures are occasionally required. Postablation increase in sinus rhythm QRS duration, RV activation time or RBBB did not translate into clinically significant RV dysfunction or worsening tricuspid valve function.

Perspectives **COMPETENCY IN MEDICAL KNOWLEDGE:** RV endocavitary arrhythmias are rare and commonly develop in structurally normal hearts as PVCs. PVCs with shorter CIs and arising from the lateral MB are more likely to degenerate into VF. Catheter ablation is an effective management strategy.

**TRANSLATIONAL OUTLOOK:** Additional research is needed to understand the anatomical and electrophysiological properties of PVCs originating in the lateral MB vs the body, including its relationship with.

## Funding support and author disclosures

The authors have reported that they have no relationships relevant to the contents of this paper to disclose.
